# Identification of Cerebrospinal Fluid Leakage by Cisternography in Recurrent Meningitis Associated With Inner Ear Malformation

**DOI:** 10.7759/cureus.113996

**Published:** 2026-08-05

**Authors:** Sota Oshika, Kayoko Kabaya, Kazuho Moribe, Shinichi Iwasaki

**Affiliations:** 1 Department of Otolaryngology–Head and Neck Surgery, Nagoya City University Graduate School of Medical Sciences, Aichi, JPN; 2 Department of Otolaryngology–Head and Neck Surgery, Ichinomiya Municipal City Hospital, Aichi, JPN

**Keywords:** cerebrospinal fluid leakage, cisternography, inner ear malformation, inner ear obliteration, recurrent meningitis

## Abstract

Cerebrospinal fluid (CSF) leakage is a recognized cause of recurrent meningitis, and accurate identification of the CSF leakage site is essential for definitive surgical management. We report an adult case of recurrent meningitis associated with bilateral congenital inner ear malformations. The combined use of radionuclide cisternography and computed tomography (CT) cisternography successfully localized CSF leakage to the right inner ear, and surgical obliteration of the right inner ear prevented further recurrence of meningitis.

A 46-year-old man experienced repeated episodes of bacterial meningitis accompanied by right-sided acute otitis media. He had experienced severe bilateral hearing loss since childhood and a prior episode of left-sided acute otitis media 11 months earlier. CT of the temporal bone revealed bilateral incomplete partition type I inner ear malformations. Radionuclide cisternography and CT cisternography using ¹¹¹In- diethylenetriaminepentaacetic acid demonstrated abnormal tracer accumulation in the right mastoid and contrast leakage into the inner ear, confirming CSF leakage at this site. Right inner ear obliteration was performed, and intraoperative findings revealed CSF leakage at the stapes footplate. The patient has remained free of meningitis recurrence.

This case underscores the diagnostic utility of combining radionuclide cisternography and CT cisternography for precise preoperative localization of CSF leakage in patients with congenital inner ear malformations.

## Introduction

Recurrent bacterial meningitis is frequently associated with underlying anatomical abnormalities of the cranial or cervical regions, accounting for approximately 60% of cases; among these cases, nearly 20% are attributable to congenital inner ear malformations [1]. When cerebrospinal fluid (CSF) leakage through the inner ear is suspected as the etiology of recurrent meningitis, precise localization of the leakage site is critical. However, preoperative identification remains challenging, particularly in cases of occult, intermittent, or low-flow CSF leaks from the temporal bone [2-4].

Radionuclide cisternography and computed tomography (CT) cisternography are valuable diagnostic modalities for identifying communications between the subarachnoid space and the temporal bone [5].

We report a very rare adult case of recurrent meningitis caused by CSF leakage associated with an incomplete partition type I (IP-I) inner ear malformation. Bilateral otitis media complicates the determination of the affected side; however, combined radionuclide cisternography and CT cisternography enable accurate preoperative localization, and surgical obliteration of the inner ear successfully prevents recurrence.

This case was previously presented as an oral presentation at the 126th Annual Meeting of the Japanese Society of Otorhinolaryngology-Head and Neck Surgery on May 30, 2025.

## Case presentation

A 46-year-old man presented with right-sided otalgia, fever, and headache without otorrhea. Four months earlier, he had developed left-sided acute otitis media, which was resolved after several days of oral antibiotic therapy. Three months before admission, he was diagnosed with bacterial meningitis accompanied by right-sided acute otitis media. Initial treatment consisted of intravenous ceftriaxone sodium hydrate (CTRX, 4 g/day) and vancomycin hydrochloride (VCM, 3 g/day). After Streptococcus pneumoniae was identified as the causative organism, the antibiotic regimen was changed to benzylpenicillin potassium (PCG, 24 million units/day). Following a total of 11 days of antibiotic therapy, both the right-sided acute otitis media and bacterial meningitis resolved. He had experienced severe bilateral sensorineural hearing loss since childhood and used a left hearing aid. There was no relevant family history.

Otoscopy revealed right tympanic membrane erythema and pulsatile bulging, consistent with acute otitis media. No tympanic membrane perforation was observed. Laboratory evaluation revealed leukocytosis (WBC count, 11,400/µL (reference range: 3,300-8,600/µL)) with neutrophil predominance (83.7% (reference range: 36.0%-69.5%)) and elevated C-reactive protein levels (1.17 mg/dL (reference range: <0.10 mg/dL)). Neurological examination revealed mild nuchal rigidity, whereas Kernig and Brudzinski’s signs were absent. CSF analysis revealed marked pleocytosis (3,070 cells/µL (reference range: 0-5 cells/μL)) with neutrophil predominance (92.3%) and hypoglycorrhachia (CSF glucose, 25 mg/dL (reference range: 50-75 mg/dL)), which was consistent with bacterial meningitis. Intravenous CTRX (4 g/day) was initiated, and therapy was subsequently switched to PCG (24 million units/day) after identification of Streptococcus pneumoniae.

**Figure 1:**
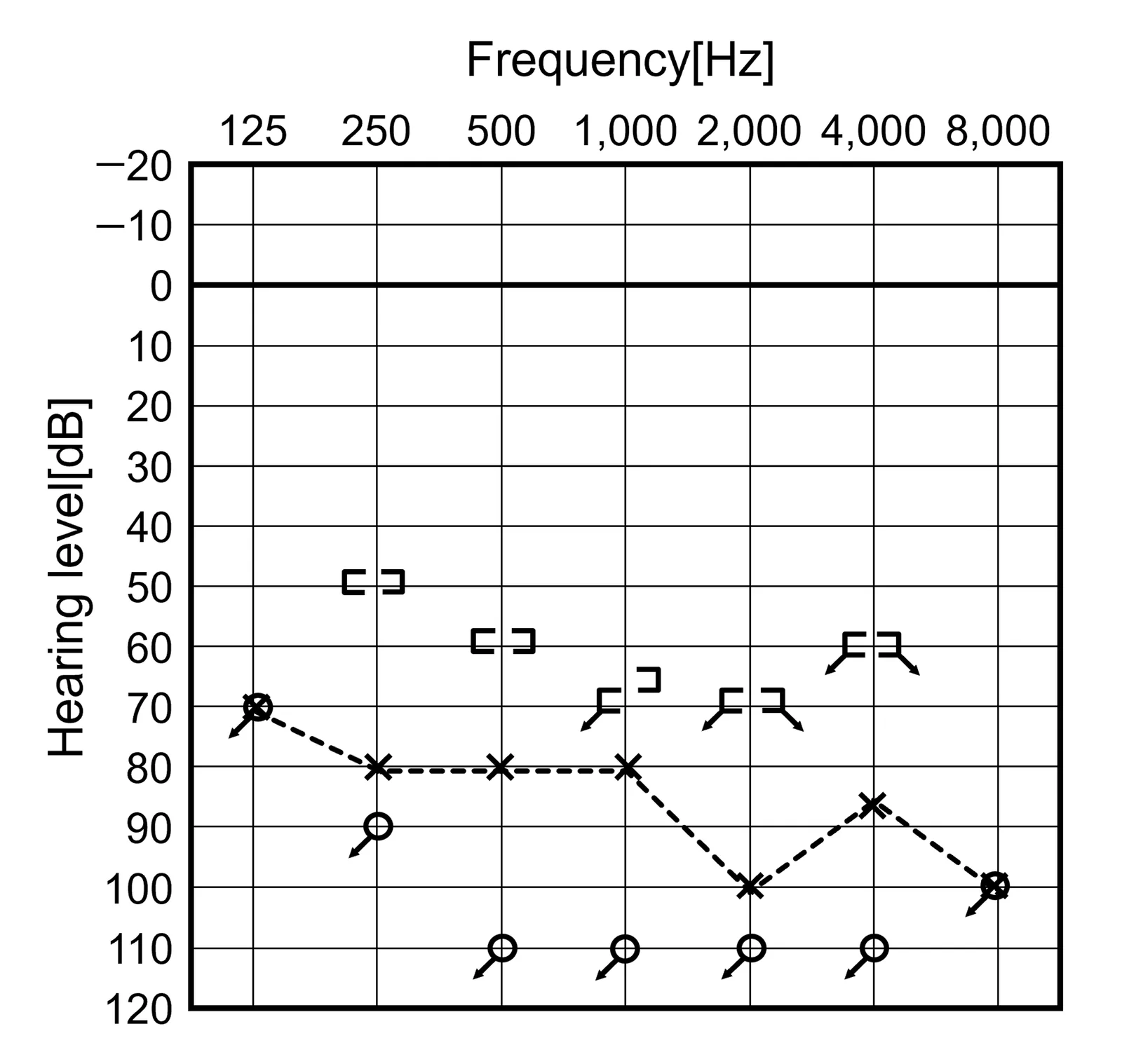
Pure-tone audiometry revealing bilateral severe-to-profound mixed hearing loss. Open circles (O) denote right-ear air-conduction thresholds, crosses (X) denote left-ear air-conduction thresholds, and brackets ([ and ]) indicate masked bone-conduction thresholds for the right and left ears, respectively. Downward arrows indicate no response at the maximum output level tested.

Given the short interval between recurrent meningitis episodes, a comprehensive otolaryngological evaluation was performed. Otoscopy revealed a pulsatile right tympanic membrane with yellow effusion, whereas the left ear appeared normal. A glucose oxidase test of the effusion collected from the right tympanic cavity was positive for glucose (2+). Cochlin-tomoprotein (CTP) testing was below the detection limit. Nasal endoscopy was unremarkable. Pure-tone audiometry revealed severe mixed hearing loss bilaterally (Figure 1). No spontaneous nystagmus was observed; however, catch-up saccades were noted during the rightward head impulse test. Caloric testing (water irrigation at 20 °C, 20 mL for 20 s) revealed right canal paresis (CP, 32.6%) with nystagmus responses lasting 157 s on the right and 79 s on the left. The fistula test was negative bilaterally.

**Figure 2:**
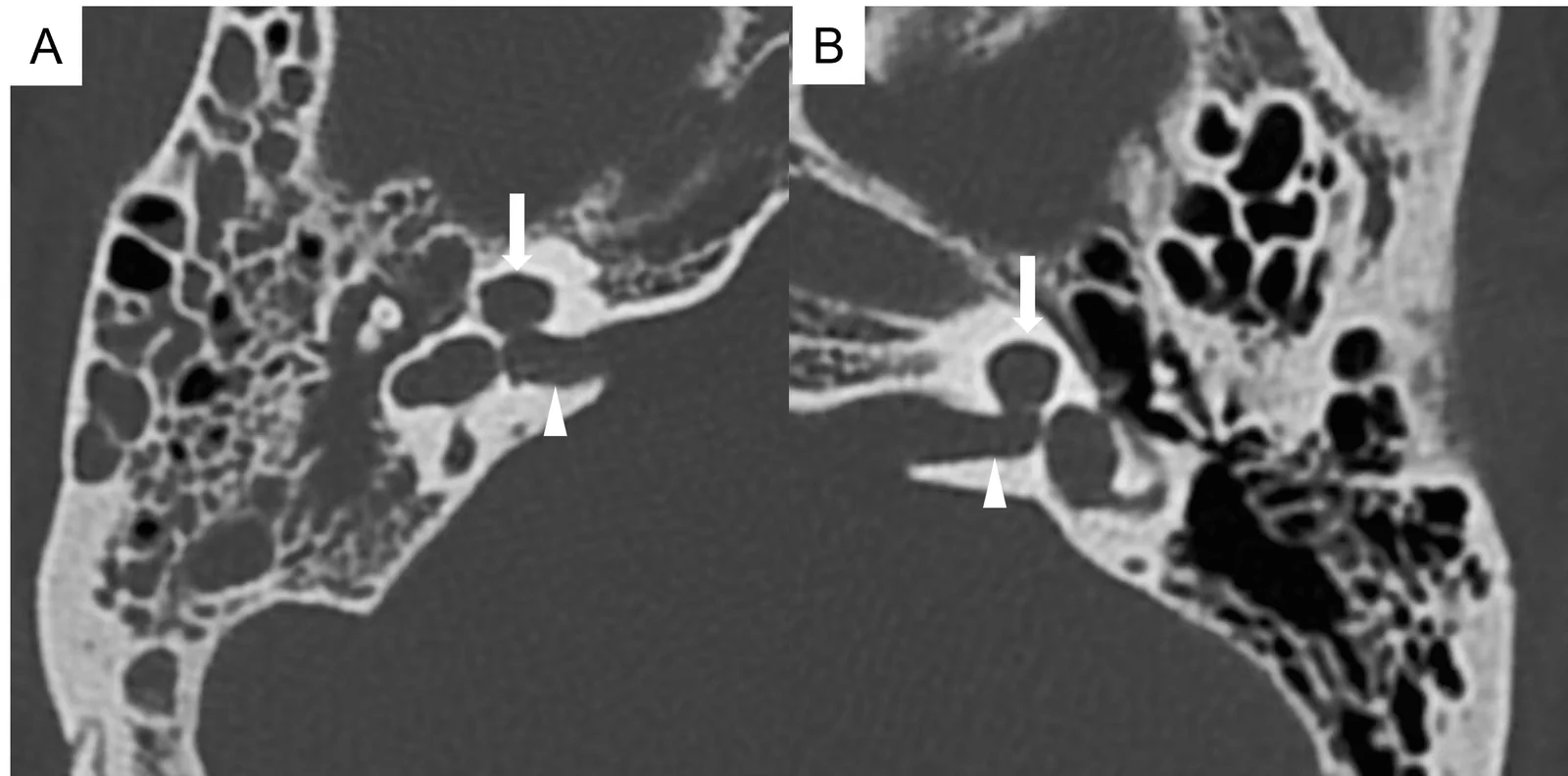
Axial CT findings of incomplete partition type I. (A) Right temporal bone: The bony partition between the internal auditory canal and the cochlea was absent, and dilation of the fundus of the internal auditory canal was observed (arrowhead). The cochlea lacked the interscalar septa and exhibited a cystic appearance (arrow). (B) Left temporal bone: Similar to the right side, dilation of the fundus of the internal auditory canal (arrowhead) and a cystic cochlea lacking the interscalar septa (arrow) were observed.

High-resolution CT (HRCT) of the temporal bone revealed increased density in the right mastoid air cells. The cochlea exhibited a cystic configuration with an absence of interscalar septa, and the bony partition between the cochlea and internal auditory canal was absent. The semicircular canals were dilated, and the vestibule was continuous with the cystically dilated cochlea on the right (Figure 2A). Similar malformations were observed on the left side (Figure 2B). These findings were consistent with bilateral IP-I inner ear malformations. Contrast-enhanced magnetic resonance imaging (MRI) revealed no intracranial abnormalities. However, it could not identify the CSF leakage site or distinguish CSF from inflammatory middle ear effusion.

**Figure 3:**
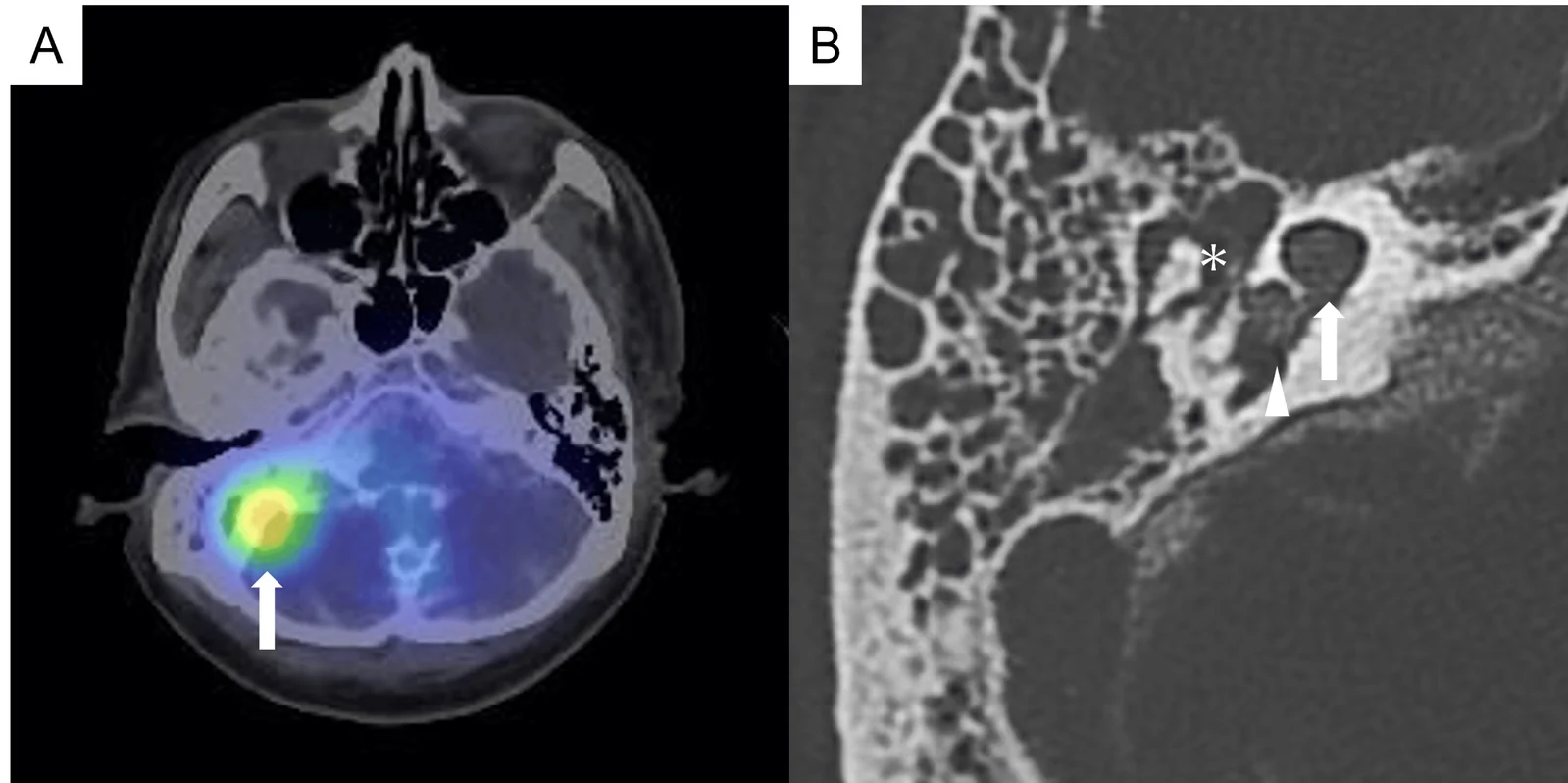
Cisternography. (A) Radionuclide cisternography: Images obtained six hours after intrathecal administration of ¹¹¹In-diethylenetriaminepentaacetic acid. Abnormal tracer accumulation was observed in the right mastoid cavity (arrow). (B) CT cisternography: Contrast leakage was observed extending from the internal auditory canal through the cochlea (arrow) and vestibule (arrowhead) into the middle ear cavity (asterisk).

To identify the affected side and localize the CSF leak, CT cisternography was performed. Following lumbar puncture, ¹¹¹In-diethylenetriaminepentaacetic acid and a nonionic iodinated contrast agent were administered intrathecally. Radionuclide cisternography revealed abnormal tracer accumulation in the right mastoid cavity (Figure 3A). CT cisternography revealed contrast leakage from the right internal auditory canal into the cochlea/vestibule and then into the middle ear cavity (Figure 3B).

Right-sided inner ear obliteration surgery was performed on hospital day 52. Intraoperatively, granulation tissue and CSF leakage were identified through a small defect in the central portion of the stapes footplate. Removal of the stapes resulted in a profuse CSF gusher, which was successfully sealed by packing the oval window with temporalis muscle and fat, reinforced with auricular cartilage. To prevent displacement of the packing, the patient was maintained on bed rest with the head elevated at 30° for three days postoperatively. The postoperative course was uneventful. The patient had preexisting profound right-sided hearing loss, and no further deterioration in hearing was observed after surgery. He experienced no postoperative vestibular symptoms, including dizziness. The patient has remained free of recurrent meningitis for 18 months after surgery.

## Discussion

We report a rare adult case of recurrent pneumococcal meningitis secondary to a congenital inner ear malformation, in which communication between the subarachnoid space and the middle ear was successfully identified preoperatively using combined radionuclide and CT cisternography. Surgical obliteration of the inner ear effectively eliminated CSF leakage.

Recurrent meningitis in patients with inner ear malformations results from congenital defects permitting abnormal communication between the subarachnoid space and the middle ear [1]. Structural abnormalities such as a dehiscent cochlear modiolus or an incompletely formed stapes footplate may allow CSF leakage through the oval window [6]. In our case, a small defect in the stapes footplate concealed by granulation tissue was identified intraoperatively.

Recurrent meningitis due to congenital inner ear malformations typically presents in childhood [1], with adult cases being uncommon [7]. The delayed onset in this patient may be attributable to granulation tissue overlying the stapes footplate, which may have initially prevented CSF leakage until it was disrupted by inflammation or pressure-related factors later in life.

Although advances in antimicrobial therapy have reduced mortality, the risk of neurological sequelae increases with each recurrence of bacterial meningitis [8]. Consequently, definitive surgical treatment is essential once a structural defect is identified [9]. Precise localization of the leakage site is critical, as HRCT alone may not delineate subtle CSF pathways [10,11] and MRI cannot reliably distinguish CSF from inflammatory middle ear effusions. In the present case, bilateral otitis media and residual effusion further complicated the diagnosis, necessitating adjunctive imaging.

Radionuclide cisternography is sensitive for detecting active or intermittent leakage [12], whereas CT cisternography provides precise anatomical localization relative to bony structures [10]. The combined use of these modalities enabled accurate identification of the leakage site and affected side in this complex case with bilateral inner ear malformations.

Once the leakage site is identified, labyrinthine obliteration with multilayer closure using soft tissue and cartilage is the preferred surgical approach [11]. In our patient, meticulous packing of the oval window successfully sealed the CSF gusher without the need for lumbar drainage. Cochlear implantation was not pursued because of long-standing right-sided deafness and patient preference.

This case highlights the clinical value of the combined use of radionuclide cisternography and CT cisternography for accurate preoperative localization of CSF leakage in congenital inner ear malformations.

## Conclusions

In patients with recurrent meningitis associated with otitis media, CSF leakage secondary to congenital inner ear malformation should be considered. In this case, combined radionuclide and CT cisternography provide complementary information when conventional imaging alone could not clearly identify the site of CSF leakage, enabling accurate preoperative localization of the leakage pathway and facilitating surgical obliteration of the inner ear. Although this experience suggests that the combined imaging approach may be useful in selected cases, larger studies are needed to validate its broader diagnostic utility.
